# Evaluation of crown-root angulation of lateral incisors adjacent to palatally impacted canines

**DOI:** 10.1186/s40510-015-0074-0

**Published:** 2015-02-26

**Authors:** Georgios Kanavakis, Karen M Curran, Kevin C Wiseman, Nicholas P Barone, Matthew D Finkelman, Sreedevi Srinivasan, Moonyoung B Lee, Caroll-Ann Trotman

**Affiliations:** Department of Orthodontics and Dentofacial Orthopedics, Tufts University, School of Dental Medicine, 1 Kneeland Street DHS#1145, Boston, MA 02111 USA; Private Practice, Boston, USA; Department of Academic Services, Tufts University School of Dental Medicine, Boston, MA USA; Department of Orthodontics, University of Iowa, Iowa City, IA USA

**Keywords:** Palatally impacted canines, Panoramic images, Lateral incisor morphology, Crown-to-root angulation

## Abstract

**Background:**

The objective of this study is to explore differences in crown-to-root angulation between lateral incisors adjacent to palatally impacted canines (PICs) and lateral incisors adjacent to normally erupted canines (NECs).

**Methods:**

Orthodontic records of 100 subjects (51 with PICs and 49 with NECs) were reviewed. Crown-to-root angulations of all lateral incisors were measured manually on the final panoramic radiographs. Also, three experienced orthodontists were asked to visually inspect the morphology of the lateral incisors on the panoramic radiographs. A mixed model was used to test the difference in crown-to-root angulation of the lateral incisor between the experimental and the control groups. The association between the examiners' observations and the presence of a canine impaction was assessed by means of a chi-square test. All analyses were performed at the 0.05 level of statistical significance.

**Results:**

A significant (*p* = 0.009) difference of 2.3° in crown-to-root angulation was found between groups. Also, 66.7% of the lateral incisors that were identified as “abnormal” by the panel of orthodontists were adjacent to a PIC. A percentage of 65.2 of lateral incisors that were identified as “normal” were located adjacent to NECs.

**Conclusions:**

The root of lateral incisors adjacent to PICs is angulated more mesially compared to lateral incisors adjacent to NECs. In addition, clinicians are somewhat able to predict if a canine is palatally impacted by visually observing the crown-to-root angulation of the adjacent lateral incisor. Evaluating the crown-to-root angulation of a lateral incisor on a panoramic image might facilitate an early diagnosis of palatally impacted canines.

## Background

Impacted canines are a frequently encountered problem in orthodontics, and the maxillary canines are the second most frequently impacted teeth after the third molars with a prevalence rate that ranges between 1% and 3% [1-4]. Canines can be impacted labially or palatally, and in non-Hispanic white populations, palatally impacted canines are at least twice as prevalent as labially impacted canines [5,6]. Moreover, palatal canine impactions are more common in females than in males with a 2:1 ratio [7], and their occurrence is bilateral in 19% to 45% of all cases [8-10]. While labial canine impactions are most often associated with the presence of maxillary crowding [6,11], 82% to 85% of palatal impactions occur in the absence of crowding.

There are two primary theories for the development of palatally impacted canines: the guidance theory and the genetic theory. The guidance theory suggests that the eruption of the canine is influenced by local factors such as a retained primary canine and/or absence, underdevelopment, or malpositioning of the maxillary lateral incisor [9,12-14]. Conversely, the genetic theory suggests that the impaction is due to a genetic predisposition; it is supported by evidence revealing an association between palatally impacted canines and other phenotypic dental variations of genetic origin such as small lateral incisor crown size [5,15,16], agenesis of lateral incisors [5,16], aplasia of premolars [16] and third molars [17], distal displacement of mandibular second premolars [18], and tooth transposition [5]. Thus, although there is no consensus about the exact etiology of palatally impacted canines, it appears that the adjacent lateral incisor demonstrates an important role, either because its eruption and dimensions are controlled by the same genes that control the eruption of the canine (genetic theory) or because its position in the arch influences the eruption path of the canine (guidance theory).

To this end, the primary aim of this retrospective, observational study was to explore the difference in the morphology of lateral incisors, specifically their crown-to-root angulation, when they are adjacent to palatally impacted canines compared with the morphology of lateral incisors adjacent to canines with a normal eruptive pattern. It was hypothesized that lateral incisors adjacent to palatally impacted canines will present with greater deviations in the crown-to-root angulation compared to lateral incisors adjacent to normally erupted canines. A secondary aim was to explore whether a subjective visual evaluation of the crown-to-root angulation of a lateral incisor on a panoramic radiograph could aid the clinician in predicting the presence of an adjacent palatally impacted canine.

## Methods

### Study population

Upon approval by the Institutional Review Board at (…) (IRB #10242), a comprehensive search of all patient electronic records was conducted at (…) using the keywords “palatally impacted canine” or “palatally impacted cuspid.” In order to be included in the study, subjects had to have at least one palatally impacted canine and have complete initial and final orthodontic records including digital pre- and posttreatment panoramic radiographs. Subjects were excluded if they had one or more missing lateral incisors, a labially impacted canine, or poor-quality radiographs. The search resulted in 51 subjects who met the inclusion criteria for the study group. The control group was composed of 49 subjects, selected consecutively from the electronic archives (2005 to 2011) of the orthodontic clinic at (…), with normally erupted maxillary canines and who had completed orthodontic treatment with full initial and final records. Prior to data collection, records were de-identified and no patient information was revealed. All panoramic X-rays were taken with the same orthopantomogram (Planmeca ProMax®, Planmeca Inc, Roselle, IL, USA).

The primary focus of this study was to evaluate the morphology of lateral incisors next to palatally impacted canines, and thus, each maxillary canine was considered as an individual sample. Therefore, if a subject had two palatally impacted canines, there were two study samples. Similarly, in a subject with normally erupted canines, there were two control samples. In those subjects with a unilateral palatal canine impaction, there was one study sample - the contralateral side was not regarded as a control because genetic predisposition could influence the morphology of the lateral incisor. As a result, there were a total of 175 canines (77 impacted, 98 normal). Subsequently, seven additional canines were excluded due to unclear root morphology of the adjacent lateral incisor on the panoramic radiograph. Therefore, the final study sample consisted of 168 canines (70 impacted, 98 normal).

### Methodology

All final (posttreatment) digital panoramic radiographs were de-identified, printed, and given a random code in order to eliminate examiner bias. Only one investigator (SS) had access to the identified subject information and was not part of the data collection or analysis.

#### Measurement of the crown-to-root angulation of the lateral incisor

**Figure 1:**
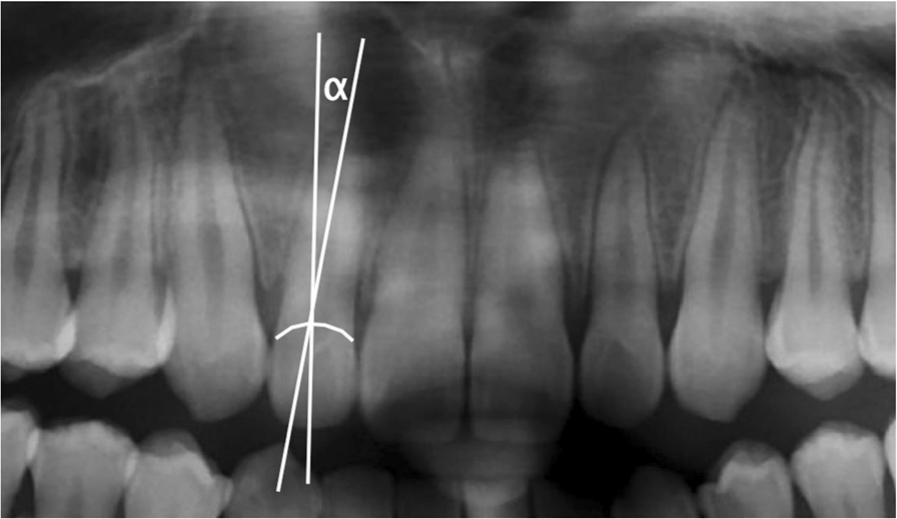
**Angle “α” was used to measure mesio-distal crown to root angulation of the lateral incisors.**

**Figure 2:**
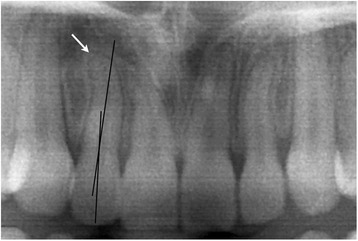
**Dilacerations at the root apex were not regarded when defining the long axis of the root.**

**Figure 3:**
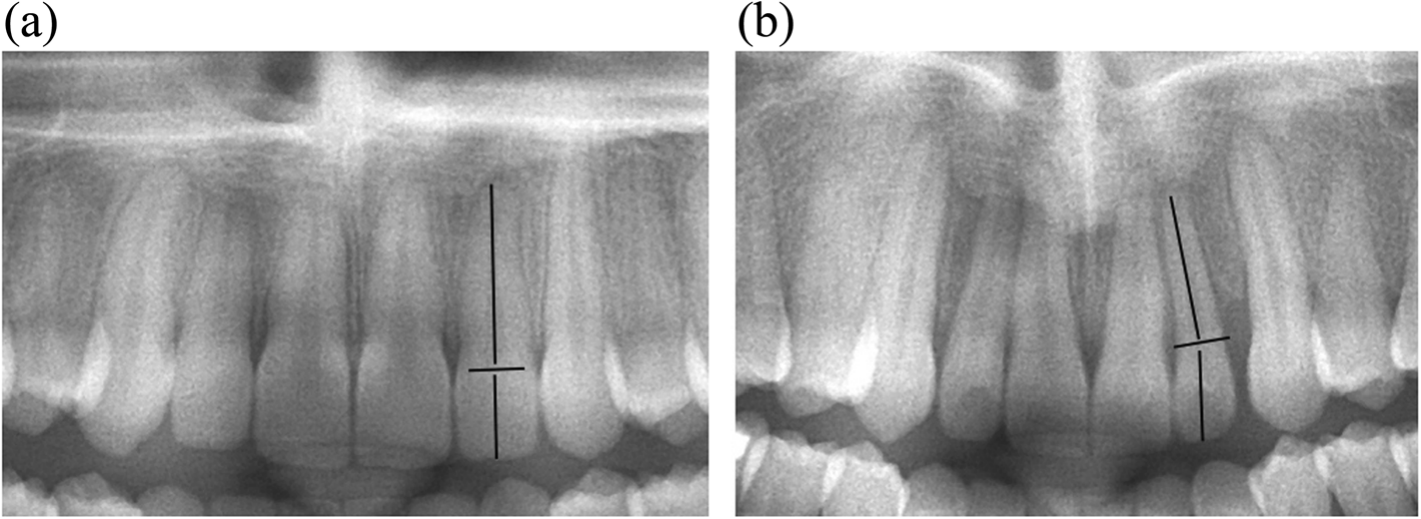
**Examples of (a) a “normal” (α-angle = 0) and (b) medially angulated lateral incisor (α-angle > 0).**

Manual measurements of the angle (degree measurement) between the long axis of the crown and the long axis of the root of all maxillary lateral incisors were performed on the final panoramic radiographs (Figure 1) by two investigators (KC, KW), separately. When defining the long axis of the lateral incisor root, dilaceration at the root apex was not considered so as to obtain a better representation of the direction of the long axis (Figure 2). When the long axis of the root was angulated mesially compared to that of the crown, a positive degree measurement was recorded, and when the angulation was distal, a negative measurement was recorded (Figure 3). In order to reduce random error, measurements from both examiners were averaged into a single value.

#### Visual evaluation of maxillary lateral incisors

Hard copies of all de-identified final panoramic radiographs were randomized and evaluated separately by three orthodontists (CC, CP, and DC), each of whom had at least 20 years of clinical experience. Each asked to visually evaluate all maxillary lateral incisors and determine whether the crown-to-root angulation appeared “normal” or “abnormal.” The orthodontists were blinded to the purpose and the details of this investigation.

### Statistical analysis

A power calculation was conducted using nQuery Advisor (Version 7.0). In order to assess the power of the study, a pilot study was performed using data from 18 subjects who were not included in the final sample population. Based on the results of the pilot study, the estimated mean difference between the two groups was 4°, with a common standard deviation of 3.5°. Using these estimated values, a sample of 49 subjects with lateral incisors adjacent to normal canines and 51 subjects with at least one lateral incisor adjacent to a palatally impacted canine was adequate to obtain a type I error rate of 5% and a power > 99%.

For the crown-to-root angulation measurement, inter-rater reliability between the two operators (KC, KW) was assessed via the method of Bland-Altman [19] prior to using the averaged measurements.

To test for potential differences in crown-root angulation between lateral incisors adjacent to palatally impacted canines and lateral incisors adjacent to normally erupted canines, a mixed model was used. The dependent variable of the mixed model was the angular measurement; the presence or absence of palatal impaction was defined as a fixed effect, and the subject was defined as a random effect. This model was selected in order to account for the dependency of results within a given subject.

To evaluate the visual assessment of the three orthodontists, their individual answers were combined into a single binary variable based on majority agreement. A chi-square test was used to evaluate the association between the examiners' visual observations of the lateral incisor and the actual presence of a palatally impacted canine. In addition, the relationship between the examiners' observations and the crown-to-root angulation of lateral incisors was assessed via Student's *t* test for independent samples. All statistical analyses were performed at the 0.05 level of significance.

## Results

**Figure 4:**
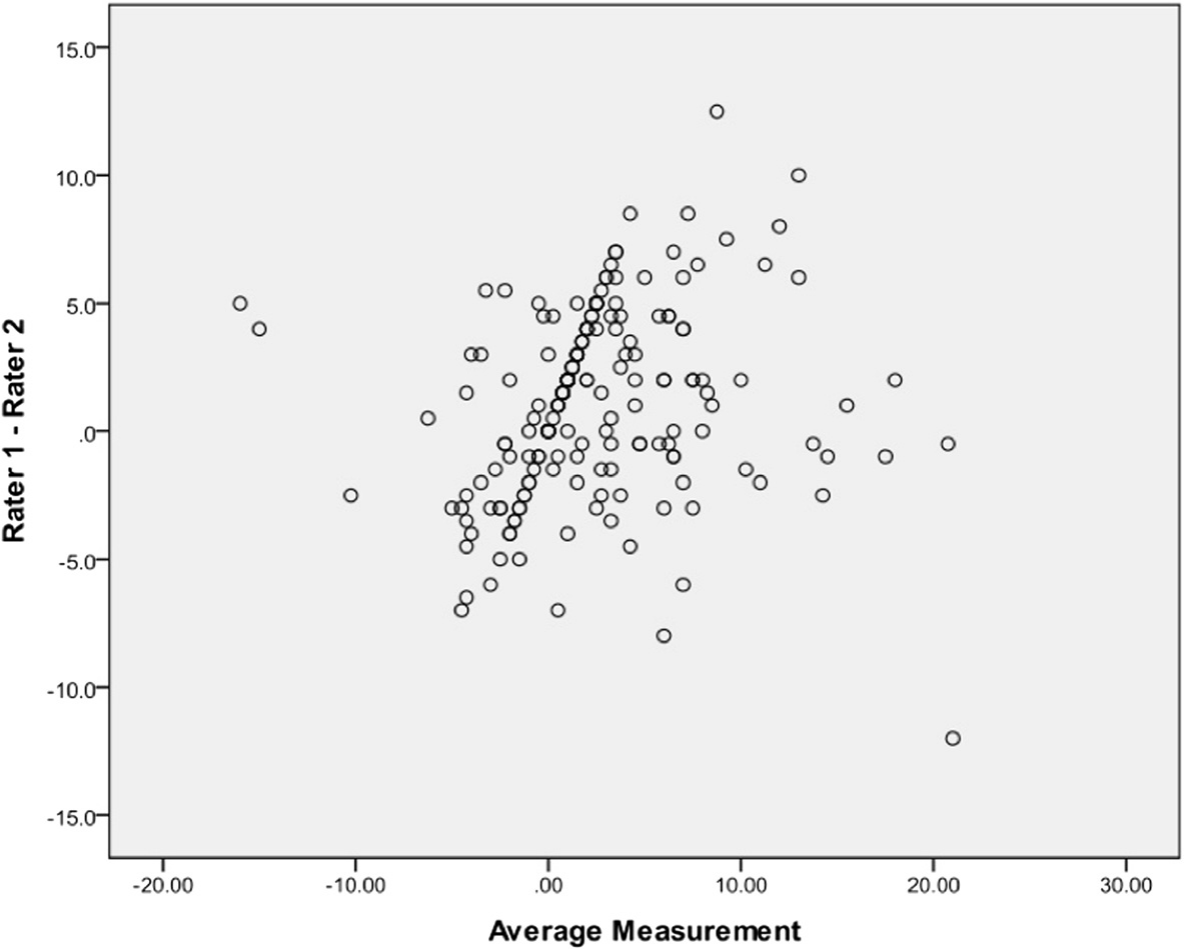
**Bland-Altman graph displaying the inter-rater reliability in measurements.**

The results for the Bland-Altman plot for inter-rater reliability are presented in Figure 4. The average difference in measurement between the two investigators was 3°; however, in a few instances, this difference was as high as 12°. In order to reduce random error, the average measurement of the two investigators was used for all statistical analyses.

Descriptive statistics (mean and standard deviation) for crown-root angulation of lateral incisors are shown in Table 1. Overall, the results revealed that the roots of lateral incisors were on average 2.30° (*p* = 0.009) more mesially angulated when the lateral incisor was adjacent to a palatally impacted canine (Table 1).

**Table 1:** **Comparison of impacted and non-impacted canines in terms of the adjacent lateral incisor's crown-root angulation**

	**Canine impaction**	***N***	**Mean**	**SD**	***p*** **value**
Crown/root angulation (º)	Not impacted	98	1.30	4.39	0.009
	Impacted	70	3.60	5.99	

**Figure 5:**
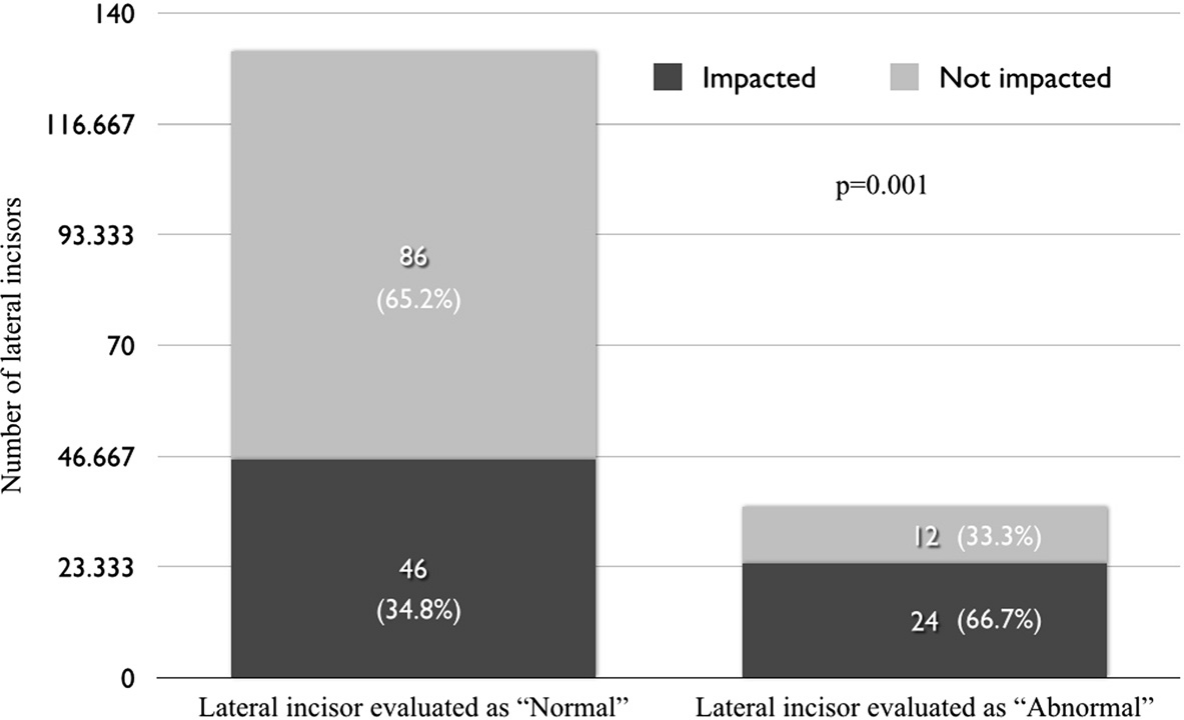
**Direct comparison of examiners' opinions and actual presence of canine impaction.**

Results for the secondary outcome of this study revealed that when experienced orthodontists described a lateral incisor as “abnormal”, its adjacent canine was palatally impacted in 66.7% (24/36) of cases. In cases when a lateral incisor was described as “normal” by the orthodontists, the adjacent canine had erupted normally in 65.2% (86/132) of the cases. The association between the orthodontists' observations and the actual presence of a palatally impacted canine was statistically significant (*p* = 0.001). Detailed results for this comparison are displayed in Figure 5. Furthermore, the mean crown-to-root angulation was significantly higher when the lateral incisors were considered to be “abnormal” by the orthodontists, compared to lateral incisors considered to be “normal” (*p* = 0.002) (Table 2).

**Table 2:** **Comparisons between the examiners' evaluations and the lateral incisor crown-root angulation measurements**

	**Examiners' consensus**		**Mean**	**SD**	***p*** **value**
Crown/root angulation (º)	Normal lateral incisor	132	1.21	3.22	0.002
	Abnormal lateral incisor	36	6.08	8.51	

## Discussion

The present investigation tested the assumption that the crown-to-root angulation of lateral incisors adjacent to palatally impacted canines differs compared to lateral incisors adjacent to normally erupted canines. The results indicated that when a canine is palatally impacted, the long axis of the adjacent maxillary lateral incisor tends to be angulated more mesially by approximately 2.5° when compared to lateral incisors with normal adjacent canines. This difference was found to be statistically significant.

Furthermore, a close look at the data reveals a trend in the morphology of lateral incisors adjacent to palatally impacted canines. In subjects where the root of the lateral incisor was angulated more than 6° mesially to the crown, 48% (20/42) of the adjacent canines were palatally impacted. This percentage increased to 68% (15/22) when the crown-to-root angulation was greater than 7.5°. In three cases with extreme crown-to-root angulation of the maxillary lateral incisor (greater than 18°), the adjacent canine was always (100%) palatally impacted. These observational findings suggest that there might be a diagnostic value to the crown-to-root angulation of the maxillary lateral incisor.

Previous studies have reported significant associations between the morphology of lateral incisors and the presence of a palatally impacted canine. Liuk et al. [20] compared the dimensions of lateral incisors in cases with palatally impacted canines to normal controls using cone-beam-computed tomography (CBCT) and revealed that the former exhibited significantly smaller crown and root dimensions. Morphologically abnormal maxillary lateral incisors have been associated with palatally impacted canines by numerous investigators [5,8,10,15,21,22]; however, there is no consensus regarding the scientific reasoning for this observation. Some tend to support that an abnormally shaped, peg, or missing lateral incisor will cause the adjacent canine to impact by not guiding it into the correct position in the arch [6,12,23,24]. On the other hand, there are numerous studies suggesting that abnormally shaped lateral incisors and palatally impacted canines are both phenotypic expressions of specific genes and therefore tend to occur concomitantly [7,17,21,25]. Results from the present investigation could potentially be used to support either of the two prevailing theories. In support to the guidance theory, it could be assumed that a mesially angulated root would not provide appropriate guidance for the eruption of the adjacent canine. At the other end of the spectrum, the same morphological discrepancy could also be considered a developmental abnormality of genetic origin.

This study also found that orthodontists tended to be able to “predict” the presence of a palatally impacted canine by observing the adjacent lateral incisor. When the lateral incisor was considered to be “abnormal,” 66.7% of the adjacent canines were palatally impacted. Similarly, in 65.2% of normally erupted canines, the consensus of the orthodontists dictated that the adjacent lateral incisor appeared “normal” (Figure 5). In addition, the description of a lateral incisor as “abnormal” was significantly associated with an increased mesial angulation of its root (Table 2). These findings suggest that the crown-to-root angulation of lateral incisors, as seen on a panoramic radiograph, might have a predictive value when a clinical decision has to be made regarding possible palatal displacement of the adjacent canine. As suggested previously, early diagnosis can lead to prevention of future palatal impaction, if appropriate treatment modalities are used [2,4,26].

A limitation of the present study is associated with the use of panoramic radiographs to determine crown-to-root angulation of lateral incisors. Previous research has suggested that measurements on panoramic radiographs tend to overestimate the mesial angulation of lateral incisors when compared to a three-dimensional image (CBCT) [27]. In addition, there is an inherent error in using a two-dimensional image to depict three-dimensional structures since the bucco-distal tooth angulations might influence mesio-distal measurements on the panoramic radiographs [28]. Possible gender and age dimorphism in lateral incisor crown-to-root angulation could have also impacted the results of this investigation. This could be a question for future epidemiological research projects. Despite these limitations, there still is substantial clinical value to the findings of this study, especially because the panoramic radiograph is still the most commonly used radiograph in dentistry. Future investigations using computed tomography should be conducted to further clarify the findings of this investigation.

## Conclusions

1. When measured on a panoramic radiograph, the root of maxillary incisors adjacent to palatally impacted canines is more mesially angulated to the crown, compared to lateral incisors adjacent to normally erupted canines.
2. Experienced orthodontists are able to “predict” the presence of a palatally impacted canine in two out of three cases, by observing the maxillary lateral incisor.
